# Lack of adrenomedullin in mouse endothelial cells results in defective angiogenesis, enhanced vascular permeability, less metastasis, and more brain damage

**DOI:** 10.1038/srep33495

**Published:** 2016-09-19

**Authors:** Laura Ochoa-Callejero, Andrea Pozo-Rodrigálvarez, Ricardo Martínez-Murillo, Alfredo Martínez

**Affiliations:** 1 Oncology Area, Center for Biomedical Research of La Rioja (CIBIR), C/Piqueras 98, 26006-Logroño. Spain; 2 Neurovascular Research Group, Department of Molecular, Cellular and Developmental Neurobiology, Cajal Institute, Av. Doctor Arce 37, 28002-Madrid. Spain

## Abstract

Adrenomedullin (AM) is a vasodilating peptide involved in the regulation of circulatory homeostasis and in the pathophysiology of certain cardiovascular diseases. AM plays critical roles in blood vessels, including regulation of vascular stability and permeability. To elucidate the autocrine/paracrine function of AM in endothelial cells (EC) *in vivo*, a conditional knockout of AM in EC (AM^EC-KO^) was used. The amount of vascularization of the matrigel implants was lower in AM^EC-KO^ mice indicating a defective angiogenesis. Moreover, ablation of AM in EC revealed increased vascular permeability in comparison with wild type (WT) littermates. In addition, AM^EC-KO^ lungs exhibited significantly less tumor growth than littermate WT mice using a syngeneic model of metastasis. Furthermore, following middle cerebral artery permanent occlusion, there was a significant infarct size decrease in animals lacking endothelial AM when compared to their WT counterparts. AM is an important regulator of EC function, angiogenesis, tumorigenesis, and brain response to ischemia. Studies of AM should bring novel approaches to the treatment of vascular diseases.

Adrenomedullin (AM) is a 52 amino acid peptide, in humans, that belongs to the amylin/calcitonin gene-related peptide family. AM is synthesized as part of a larger precursor molecule, termed preproadrenomedullin. This precursor consists of 185 amino acids in humans. Preproadrenomedullin contains a 21-amino acid N-terminal signal peptide that immediately precedes a 20-amino acid amidated peptide, designated proadrenomedullin N-terminal 20 peptide or PAMP. AM exerts its actions through combinations of the calcitonin receptor like receptor or CLR; and either receptor activity-modifying protein 2 (RAMP2) or RAMP3 (known as AM1 and AM2 receptors respectively). AM is a vasoactive peptide involved in the regulation of circulatory homeostasis and in the pathogenesis of certain cardiovascular diseases^1^. AM is constitutively secreted by vascular endothelial cells (EC) and smooth muscle cells and is expressed at a high level in the lung^2^. AM plays critical roles in blood vessels, including regulation of vascular stability and permeability^3^. Previous studies described that embryos lacking AM signaling (AM−/−) die in utero due to leaky and unstable blood^3^ and lymphatic vessels^4^. Mice partially lacking AM develop cardiovascular abnormalities, including overdeveloped ventricular trabeculae and underdeveloped arterial walls^3^. Therefore, AM is required for the development and/or maintenance of the vasculature during embryogenesis. Besides, AM is also essential for angiogenesis^5^. AM is also involved in the regulation of inflammation and cell survival. Blood vessel-specific AM transgenic mice showed that AM exerts anti-atherogenic and organ protective effects under various pathophysiological conditions^6^,^7^.

Vascular EC and vasoactive molecules play key roles in the maintenance of vascular and organ homeostasis^8^,^9^,^10^,^11^. They actively secrete a variety of bioactive molecules, including nitric oxide, atrial natriuretic peptide, prostacyclin, and AM, among many others. AM has attracted much attention as it also exerts diuretic and cardiotonic effects, and is involved in the regulation of hormone release, cell survival, inflammation, and oxidative stress^1^. Plasma levels of AM are elevated in patients with cardiovascular diseases such as hypertension, congestive heart failure, and myocardial infarction^12^,^13^,^14^. Moreover, it was reported that plasma AM levels are a highly sensitive marker of chronic kidney disease that may have prognostic value^15^.

AM has critical roles in tumor cell growth and cancer invasiveness, and is involved in tumor angiogenesis through promotion of recruitment of hematopoietic progenitors, vascular morphogenesis, and blood vessel stabilization and maturation. Inhibition of AM has antitumoral effects linked to antiangiogenic effects but in some cases it also has direct antiproliferative activity on cancer cells^16^. There are now many studies that describe an association between AM expression and cancer^17^. Over the last years, numerous authors have reported the regulatory properties that AM possesses on the proliferation of a wide variety of cancer cells^18^,^19^. Many studies support the idea that AM functions as a potent autocrine/paracrine growth factor for tumor cells and demonstrate that reduction of endogenous AM can potentially impair tumor growth *in vivo*^17^. Various studies using human tumor xenografts in immuno-deficient mice have shown that lowering AM levels reduces tumor growth^20^,^21^,^22^,^23^.

AM affects not only tumor cells, but also EC in the surrounding microenvironment. AM was also localized to the EC of the surrounding stroma^24^. Since AM directly impacts EC proliferation and permeability, AM induced modulation of vessels may affect the spread of cancer cells to distant sites via blood or lymphatic vasculature.

AM is produced by many areas of the central nervous system^25^ and peripheral tissues^1^. AM expression is also upregulated by hypoxia through activation of the HIF-1 pathway^19^ and it has been shown to increase following ischemic insults to the brain^26^,^27^, including ischemic stroke in patients^28^. Many articles in the literature ascribe a neuroprotective role to AM^26^,^27^,^29^. Studies in mice lacking AM expression suggest that this peptide is neuroprotective in the context of stroke^30^. Nevertheless, high AM levels in stroke patients associate with poor prognosis^31^.

To clarify the pathophysiological function of vascular AM, we generated a conditional EC-specific knockout (KO) mouse model (AM^EC-KO^). In contrast to E-RAMP2−/− neonates, the AM^EC-KO^ neonates appeared healthy. Apparently, the phenotype caused by knocking out the ligand during development is much milder than knocking out the receptor side^32^. This is likely because AM is secreted from cells other than EC, and circulating and/or paracrine AM from these other sources may compensate for the endothelial AM deficiency during development. In adults, however, the chronic reduction in AM signaling led to vascular inflammation, vascular senescence, chronic inflammation, enhanced oxidative stress, organ fibrosis, and chronic organ damage, suggesting that aging is another important factor contributing to the emergence of the pathophysiological phenotype^32^.

Here we demonstrate that mice lacking AM in their EC had defective angiogenesis *in vivo* and increased vascular permeability in comparison with wild type (WT) littermates. In addition, we tested the impact of the lack of AM on two disease models where vascular AM may play important roles: lung cancer metastasis and brain focal ischemia.

## Results

### Characterization of AM^EC-KO^ mice

AM^EC-KO^ model was obtained by crossing animals whose *adm* gene was flanked by LoxP sequences with transgenic mice expressing Cre recombinase under the Cdh5 promoter, which is specific of EC^32^. AM^EC-KO^ mice were born and reached weaning age at the expected Mendelian frequency. This can be shown by following their body and organ weight, which reveals no differences among genotypes (data not shown). In addition, birth rates were the same irrespective of the dam’s genotype (results not shown) indicating that lack of AM in the EC does not influence body weight or maintenance of pregnancy. However, AM^EC-KO^ females, but not males, exhibited a slight increase in mortality (2.5%) over time (24–60 w).

### Blood pressure

One of the better known functions of AM is the regulation of blood pressure^1^, so we tested how the deletion of *Adm* in EC influenced this parameter. There was a significant decrease in systolic blood pressure and an increase of tail blood volume (p < 0.001) in the KO mice whereas this change was not significant in diastolic blood pressure, pulse frequency and tail blood flow rate (Table 1).

### AM^EC-KO^ mice show reduced angiogenesis

**Figure 1:**
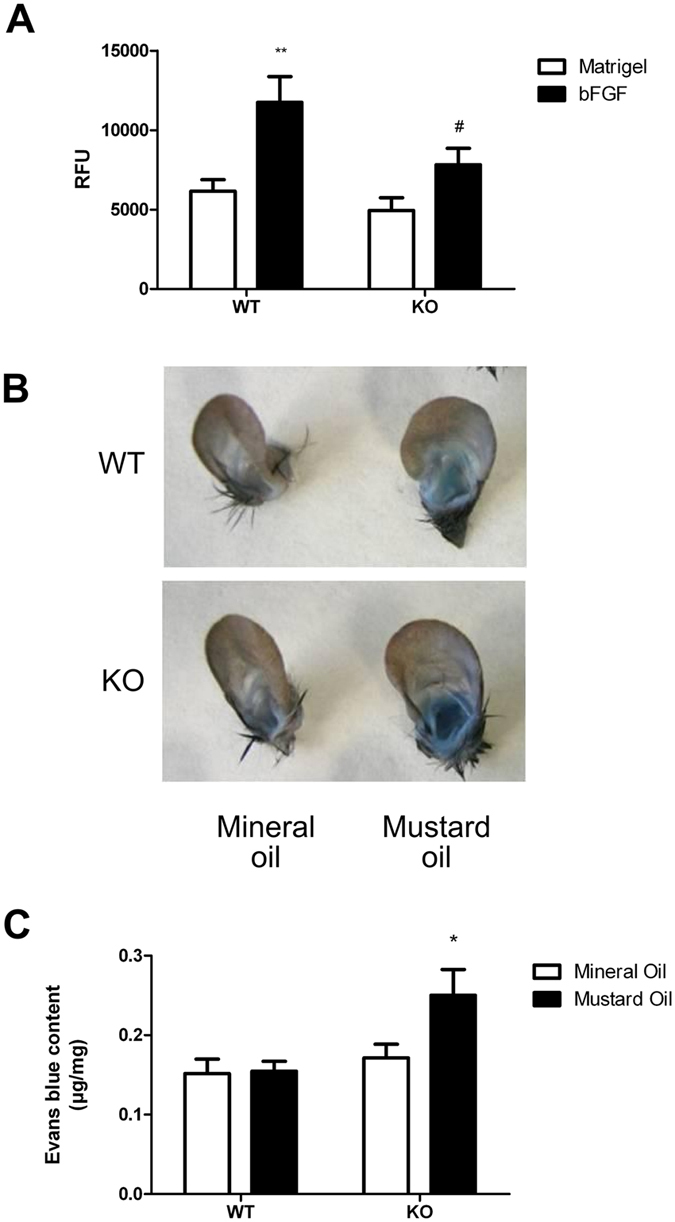
AM^EC-KO^ mice exhibit reduced angiogenesis (**A**) and enhanced EC/plasma leakiness in ear skin (**B**,**C**). (**A**) Silicone tubes containing Matrigel with or without bFGF were implanted under the skin of WT and AM^EC-KO^ mice for 11 days. The mice were then injected intravenously with 25 mg/mL fluorescein isothiocyanate (FITC)–dextran, and 20 minutes later, the tubes were removed from the skin pockets. Quantification of FITC–dextran (in arbitrary units) trapped within the implants was used as an indirect measure of the volume of blood circulating through the newly formed vessels. Implants containing serum-free DMEM (open bars) were used as a control. At least five animals (2 tubes/mouse) per group were used. **Corresponds to *p* < 0.01 with respect to Matrigel. ^#^Corresponds to *p* < 0.05 with respect to WT. (**B**,**C**) Vascular permeability in KO (n = 8) and WT mice (n = 8) was measured in response to mustard oil with Evans blue dye. (**B**) Photographs of ears after treatment with mustard oil (30 min) and vascular perfusion, showing relative amount of extravasated Evans blue tracer. Ear from a WT mouse is blue around the margin of the ear, whereas ear from a AM^EC-KO^ mouse is strongly blue throughout the ear. (**C**) Mustard oil treatment (closed bars) induced significant leakage in vessels of AM^EC-KO^ mice. Local vascular permeability was increased in KO mice (*p < 0.05) 30 min after topical application of mustard oil. No difference was observed in unstimulated mice.

We performed a quantitative *in vivo* assay to determine the angiogenic potential of mice lacking AM in EC. Open silicone tubes filled with Matrigel containing bFGF or vehicle were implanted subcutaneously in WT or AM^EC-KO^ mice for 11 days. At the end of the incubation period, the implants containing bFGF included more visible tufts of capillaries than implants with Matrigel alone. In addition, in the KO mice this increase was lower than in the WT animals. Implants containing bFGF in WT mice were significantly higher than those containing vehicle (p = 0.004), whereas there were no differences in the KO mice. Furthermore, the implants containing bFGF in the KO mice were significantly lower than those on the WT animals (p = 0.036). This observation was confirmed by quantifying the amount of FITC–dextran collected by the implants as an indirect measurement of the volume of blood circulating through the newly formed vessels. Implants from WT mice with Matrigel control were indistinguishable from the AM^EC-KO^ mice implants (Fig. 1A).

### Enhanced vascular permeability in AM^EC-KO^ mice

AM is known to regulate vascular permeability *in vitro* and *in vivo*^28^, in part by preventing endothelial contraction and intercellular gap formation. Extravasation of Evans blue dye at 15–30 minutes was much more extensive in AM^EC-KO^ mice than in WT mice (Fig. 1B). Mustard oil is a local irritant that induces plasma leakage and inflammation. However, even with mustard oil treatment, the blue coloration was less intense in the WT mice than in AM^EC-KO^ mice (Fig. 1B). The amount of extravasated dye was quantitated by spectrophotometric analysis revealing that the amount of extravasated Evans blue dye in the ears was significantly greater in AM^EC-KO^ mice than in the WT mice (Fig. 1C) (p = 0.020).

### EC from AM^EC-KO^ mice exhibit reduced proliferation and migration

**Figure 2:**
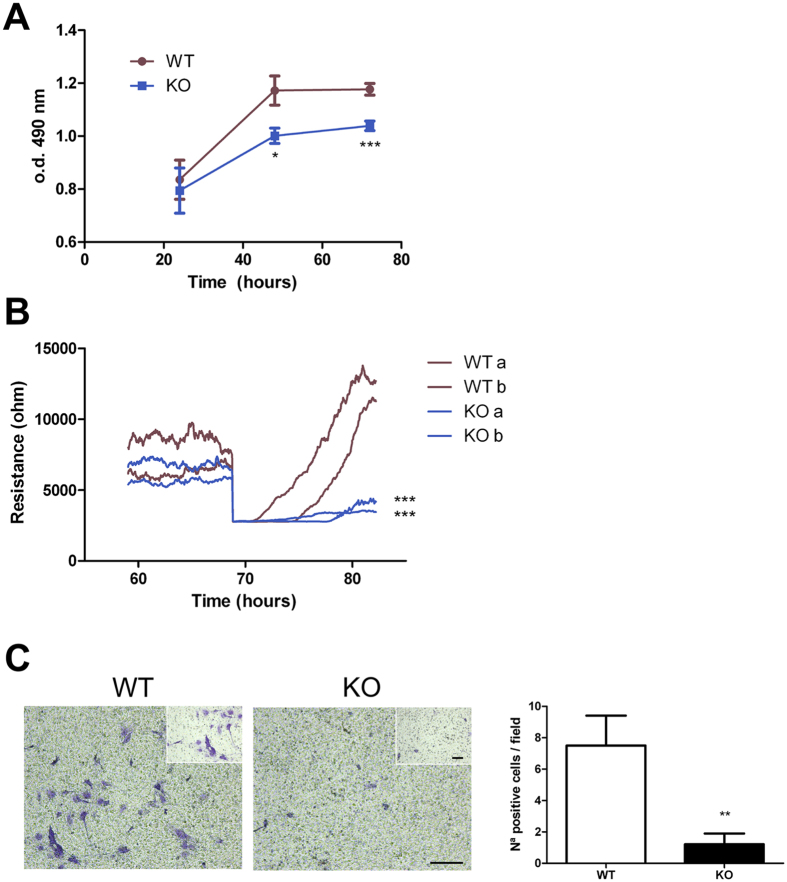
Lung EC from AM^EC-KO^ mice show reduced proliferation and migration *in vitro.* (**A**) Effect of AM on EC proliferation, as measured by MTT assay. Cells were incubated for 3 days in medium containing 20% FCS. Data represent the mean ± SEM of optical density measured at 490 nm. n = 9 for each genotype. *p < 0.05, and ***p < 0.001 with respect to WT. (**B**) Cell migration after electric wounding. Impedance recordings were performed at 4 kHz with maximal temporal resolution. MLEC were seeded on coated 8W1E arrays and grown to confluence. Then an electrical wound was created by application of one 5 V pulse at 60 kHz with a duration of 20 sec. WT MLEC showed better wound healing capabilities based on a faster wound closure (migration, slopes of the curves) in comparison to the KO MLEC. Data shown are results from 2 different cell cultures. ***p < 0.001 with respect to WT. (**C**) Transwell migration assay. WT or AM^EC-KO^ cells were seeded in the upper chamber of the transwell, conditioned medium containing 20% of FCS and ECGS was placed at the bottom side. After 48 h, migrated cells were quantified by crystal violet staining. All conditions of the experiment were performed in duplicate. At least 7 pictures were taken from each well (10x and 20x). The histogram represents the mean ± SEM of these quantifications. **p < 0.01 with respect to WT. Size bar for large photographs: 200 μm. Size bar for the inserts: 100 μm.

We examined the effect of the lack of AM on lung EC proliferation. AM^EC-KO^ cells showed lower proliferative rates than WT EC (Fig. 2A; p = 0.014 at 48 h and p < 0.001 at 72 h). In an impedance-based wound healing assay EC from AM^EC-KO^ mice migrated at significantly lower rates than their WT counterparts (Fig. 2B; p < 0.001). The effect of AM on EC migration was also assessed using transwell migration assays. As shown in Fig. 2C, AM^EC-KO^ cells showed significantly decreased cell migration compared with WT cells (p = 0.007).

### EC ultrasequencing

**Figure 3:**
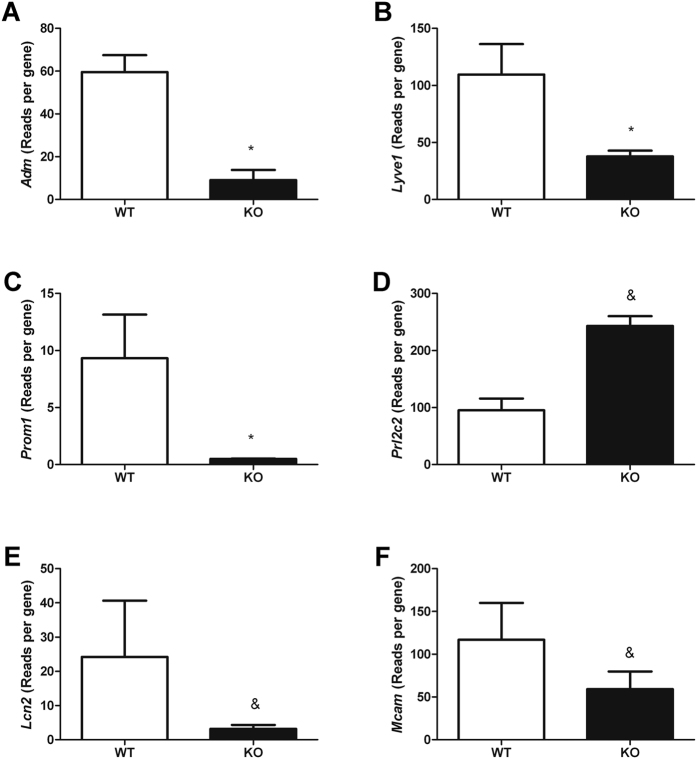
Lung EC from AM^EC-KO^ mice reveal altered gene expression. Quantification of the expression of several genes at the mRNA level by ultrasequencing. Genes were analyzed by next generation/massive sequencing and are expressed as reads per gene. Bars represent the mean ± SEM of at least 3 MLEC preparations. ^&^*p *< 0.1 and *p < 0.05 with respect to WT.

To try to better understand the differences on cell physiology observed in AM^EC-KO^ versus WT EC, we performed next-generation transcriptomic analysis of these cells. Mouse lung endothelial cells (MLEC) retained high levels of RNA expression for three endothelial markers (*Pecam-1, Icam2,* and *Cdh5*, data not shown) in culture demonstrating the quality of the cell purification method. *Adm, Lyve1, Prom1, Prl2c*2, *Lcn2* and *Mcam* were the genes that experienced larger variations after AM deletion (Fig. 3; p < 0.1). *Adm, Lyve1, Prom1, Lcn2* and *Mcam* were downregulated in EC from AM^EC-KO^ mice whereas *Prl2c*2 was upregulated in AM^EC-KO^ cells.

### Endothelial function during tumor metastasis

**Figure 4:**
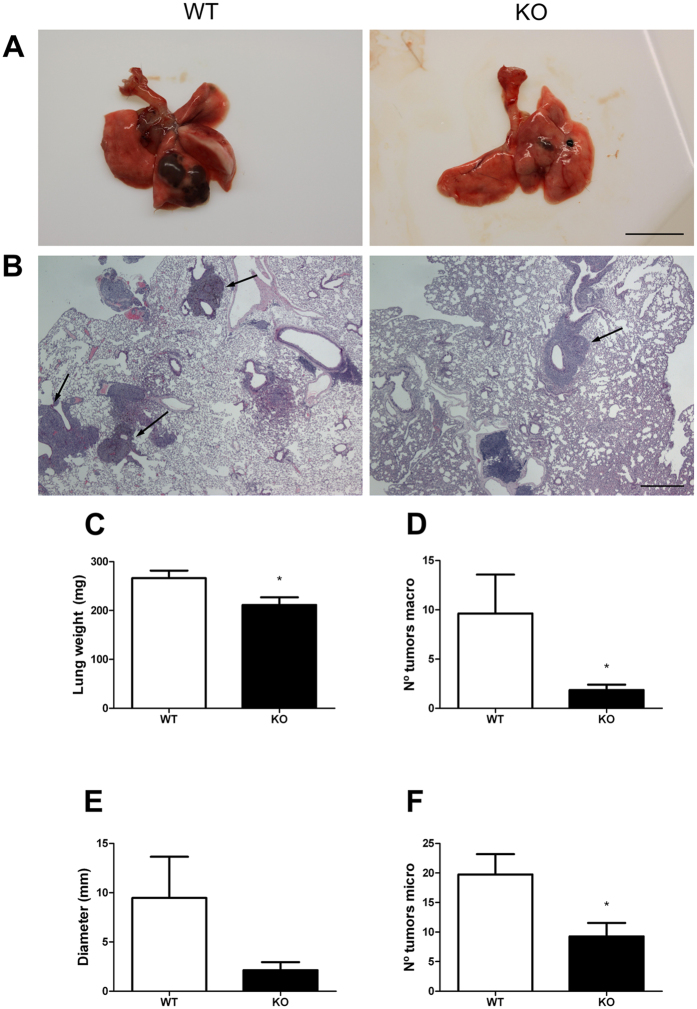
Lack of AM in EC results in reduced B16-BL6 metastatic lung disease. (**A**) Gross evaluation of lungs reveals that AM^EC-KO^ mice develop substantially less and smaller metastases after B16-BL6 melanoma injection. Images are representative of results obtained from WT and AM^EC-KO^ mice. (**B**) H&E analysis of sections obtained from the lungs shown in A. Metastases are marked with arrows. (**C**) Metastases were evaluated by change in lung weight compared with control. Total lung mass decreased in KO lungs (*p < 0.05) compared with controls (WT). Data represent animals from both mouse lines (n = 8). The number of tumors counted by the naked eye (**D**), the diameter of the largest tumor (**E**), and the number of tumors per tissue section (**F**) were also estimated *p < 0.05. Size bars: A = 10 mm; B = 500 μm.

In addition to its important vascular function, the endothelium is increasingly recognized for its critical role in tumorigenesis^33^,^34^,^35^,^36^. Although the endothelium provides nourishment to tumors, it also serves as a barrier to metastatic cells^37^. To determine whether the lack of AM compromised endothelial function during tumorigenesis, B16-BL6 metastatic melanoma cells were injected into AM^EC-KO^ and WT mice. After 21 days, AM^EC-KO^ lungs exhibited significantly less tumor growth than littermate control mice (WT), by visual inspection (Fig. 4A) and by studying histological sections (Fig. 4B). Systematic quantification (Fig. 4C–F) revealed a significant decrease (p < 0.05) in lung weight as well as in the number of tumors in KO versus controls.

### Effects of AM on brain infarct volume following focal ischemia

**Figure 5:**
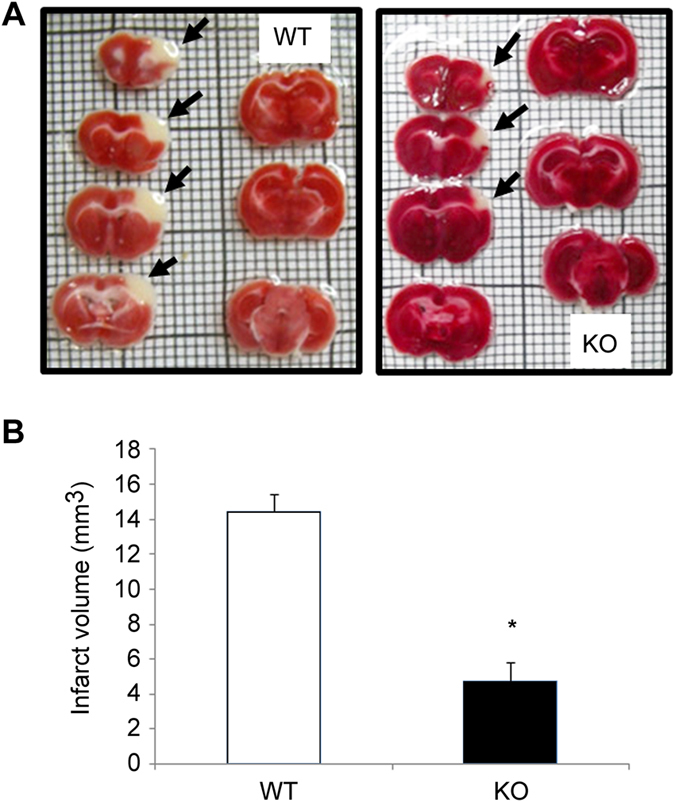
Effects of endothelial AM on MCAO injury. Deletion of AM gene in EC decreases the infarct volume after permanent focal cerebral ischemia in mice. Mice were subjected to 48 h of permanent MCAO and infarct volumes were quantified from TTC-stained serial coronal sections. Representative stacks of 7 TTC stained sections are shown for each genotype (**A**). AM^EC-KO^ mice reveal a smaller unstained area of ischemic tissue in the infarcted neocortex when compared to their WT littermates (**B**). Data are mean ± SEM, n = 4 (*p < 0.05).

AM^EC-KO^ mice and their WT counterparts were subjected to focal ischemia by permanent cauterization of the middle cerebral artery. The infarct volume, after 48 h, was significantly smaller in AM^EC-KO^ mice than in their WT littermates (Fig. 5; p = 0.031).

## Discussion

Lack of AM in EC results in changes in EC physiology and greatly influences cancer and stroke progression. AM^EC-KO^ neonates were healthy and presented no overtly pathophysiological phenotypes in contrast with mice were RAMP2, a component of the AM receptor, was eliminated from EC^32^. We suggest that in AM^EC-KO^ neonates, circulating and/or paracrine AM from sources other than EC likely attenuate the phenotypes observed in E-RAMP2−/− neonates. In the adults, however, the vascular inflammation and organ damage were milder but similar. AM^EC-KO^ showed severe infiltration and accumulation of inflammatory cells around the vasculature^32^. They also showed glomerulosclerotic changes with mesangial expansion, upregulation of inflammatory molecules and enhanced microalbuminuria^32^ indicating the importance of the autocrine effects of EC-generated AM.

Ultrasequencing results confirmed the elimination of *Adm* expression thus validating the KO model. Obviously, our construct affects the whole *Adm* gene, therefore the effects observed in this study may be due to the lack of AM, PAMP, or both.

We observed that diastolic blood pressure in the KO mice was lower than in the WT animals. This seems to run against the generalized idea which identifies AM as a vasodilator^1^. Nevertheless this issue is more complex than initially perceived. Although AM is a vasodilator when injected peripherally^38^, it acts as a vasoconstrictor when injected into the brain probably acting through vascular nerve terminals^39^. Therefore, in a EC conditional KO, the final outcome would depend on which physiological fraction, peripheral or central, plays a more relevant role on blood pressure regulation. Similar results to the ones obtained in our model have been described when RAMP2 is deleted from EC^32^ and in an inducible full body KO of AM^40^. So, it appears that the AM-regulated central control on vasodilatation is more powerful than the peripheral one.

Angiogenesis is required for the maintained growth of solid tumors and the establishment of new metastases. AM is a pro-angiogenic molecule secreted by tumors and increased by hypoxia^19^,^41^, whose inhibition results in a considerable reduction of angiogenesis and of tumor growth in animal models^24^. AM is a survival factor for cancer cells that regulates tumor initiation and progression since it has anti apoptotic actions, increases angiogenesis, proliferation and migration of tumor cells^5^. Besides, it accelerates metastatic grow^42^. Here we have shown that blocking AM in EC results in reduced angiogenesis. Altogether, these results suggest that AM directly stimulates angiogenesis, which could be one of the mechanisms by which AM contributes to cancer progression.

Vascular permeability is a critical marker for blood vessel status. Extravasation of water and small molecules is thought to occur through small openings between EC. The strength of cell-to-cell junctions is strictly regulated by interaction between molecules^43^. In our model, AM deletion in EC causes barrier dysfunction, which in turn leads to enhanced vascular permeability. It is well established that AM inhibits plasma extravasation^44^ with the postcapillary endothelium being a particular AM target^45^. AM also reduced lung permeability in an acute lung injury model of rats^46^. Overall, these data provide evidence for the role of AM as a junctional tightening factor that helps regulating EC permeability.

AM can provide tumor cells a growth advantage in addition to acting on surrounding EC to promote proliferation and changes in vessel permeability to facilitate metastasis. Mcam is part of the endothelial junction associated with the actin cytoskeleton^47^. Direct association of the actin cytoskeleton with cell adhesion proteins is essential to barrier function. Tight junctions and adherent junctions connect adjacent cells and regulate paracellular permeability^48^. If EC from KO mice do not have proper adherent junction assembly this might be the reason why KO mice do not maintain integrity of the endothelial barrier. Lyve1 is a marker of lymphatic EC^49^. Lymphatic microvasculature is an important entry point for leukocytes and tumor cells. Moreover, Prom1 is expressed in endothelial progenitor cells and contribute to the tumor vasculature in non-small cell lung cancer^50^. Besides, Lcn2 is used as a biomarker of kidney injury but also controls cell invasion and metastasis of cancer cells^51^,^52^. Therefore, downregulation of those 3 molecules (Lyve1, Prom1, Lcn2) could be responsible for lower migration capabilities of tumor (metastatic) cells in AM^EC-KO^ mice. In addition, Prl2c2 is involved in cell survival and apoptosis regulation^53^. Upregulation of Prl2c2 in samples from KO MLEC could be the mechanism by which EC from AM^EC-KO^ mice show less proliferation than EC from WT mice.

The results we obtained in the stroke model were unexpected. When AM was deleted in neurons, there was a significant increase in stroke volume when compared with WT controls indicating that neural AM exerts a neuroprotective action in the brain^30^. However, when AM is knocked out from EC, mice exhibit less ischemia-induced brain damage. Therefore, our data suggest that AM in the endothelium has a harmful effect in this context. When AM was administered systemically it showed a neuroprotective effect^54^. However, there is one paper where intracerebroventricular injection of AM resulted in an increase of ischemic injury^55^. Patients with ischemia showed increased levels of AM with worse prognosis^31^. In fact, overexpression of AM has been detected in stroke patients and this higher expression associated with stroke severity^56^. In this regard, animals deficient in adhesion molecules (such as Mcam) have decreased ischemic damage in experimental stroke^57^,^58^. Moreover, Lcn2 is a proinflammatory mediator during the acute stage of ischemic stroke^59^. Mcam and Lcn2 were decreased in AM^EC-KO^ mice, thus both molecules could be at the heart of the mechanism by which AM^EC-KO^ mice have less damage after cerebral ischemia.

In summary, vascular AM plays critical roles in the regulation of vascular integrity, including the maintenance of vascular structure, regulation of angiogenesis, and vasoprotection against vascular injury.

## Methods

### Mouse model

Conditional KO of AM in EC was achieved by crossing animals whose AM gene was flanked by LoxP sequences^60^ with transgenic mice expressing Cre recombinase under the control of the VE-cadherin promoter (Stock Number 006137, The Jackson Laboratory, Bar Harbor, Maine, USA), and has been published before^32^. All procedures were carried out in accordance with the European Communities Council Directive (86/609/CEE) on animal experiments and with approval from the ethical committee on animal welfare of our institution (OEBA-CIBIR).

### Blood pressure measurements

Blood pressure was measured in conscious animals by the tail-cuff method (CODA, Kent Scientific, Torrington, CT, USA). Briefly, each animal was acclimated for at least 10 practice sessions for a minimum of 15 minutes, then blood pressure parameters were recorded during 5 consecutive days. In each recording session, 1 set of 5 acclimation cycles followed by 3 sets of 10 acquisition cycles were performed, and the average of the last 20 successful recordings was used for calculating systolic and diastolic blood pressure, heart rate, tail blood flow, and tail blood volume.

### Directed *in vivo* angiogenesis assay (DIVAA)

Analysis and quantitation of angiogenesis was done using DIVAA as previously described^61^. Surgical-grade silicone tubes (0.15 cm outside diameter by 1 cm in length; New Age Industries, Southampton, PA, USA) were closed at one end. The lumen of each tube was filled with growth factor reduced Matrigel (BD, San Diego, CA, USA) that contained either serum-free medium or 100 μM bFGF (BD). The tubes were held at 37 °C to allow the Matrigel to solidify. Two tubes were inserted into a skin pocket in the back of each anesthetized WT or KO mouse; the pocket was sealed with surgical staples. Eleven days later, mice were injected intravenously with 25 mg/mL fluorescein isothiocyanate (FITC)–dextran (100 μL/mouse; Sigma, St. Louis, MO, USA), and 20 minutes later, the tubes were removed from the skin pockets. Matrigel was digested by incubation with dispase (BD) for 1 h at 37 °C and the amount of fluorescence trapped in the implant was measured at 485 nm using a POLARstar Omega plate reader (BMG Labtech, Ortenberg, Germany) to evaluate the volume of blood circulating through the newly formed vessels.

### Vascular permeability assay

Vascular permeability assays were performed as previously described^62^. Evans blue dye (30 mg/kg, Sigma) in 100 μl PBS was injected into the tail vein (i.v.). After 1 minute, 5% mustard oil (Sigma) diluted in mineral oil (Sigma) was applied to the dorsal and ventral surfaces of the ear with a cotton swab; the application process was repeated 15 minutes later. Photographs were taken 30 minutes after injection of Evans blue dye. After the mice were euthanized by CO_2_ inhalation, ears were removed, blotted dry, and weighed. The Evans blue dye was extracted from the ears with 1 ml formamide (Sigma) overnight at 55 °C and measured spectrophotometrically at 600 nm (BMG Labtech). Results are expressed as μg per mg of tissue.

### Isolation and culture of murine lung EC (MLEC)

The method for the isolation and purification of EC was modified from published protocols^63^. In brief, the lungs were harvested from three mice. The lung lobes were carefully dissected out from any visible bronchi and mediastinal connective tissue. Organs were washed with DMEM (Gibco, Paisley, UK) containing 20% FCS (Gibco) to remove erythrocytes, finely minced with scissors, and digested with type I collagenase (150–170 U/ml, Worthington Lakewood, NJ, USA) at 37 °C for 45 minutes. The digested tissue was mechanically dissociated by trituration, filtered through a 70-μm disposable cell strainer (BD) and centrifuged at 400 × g for 10 minutes at 4 °C. The cell pellet was resuspended in cold DPBS and incubated with PECAM-1 coated (BD) beads (Invitrogen, Carlsbad, CA, USA), at room temperature for 10 minutes with end-over-end rotation. A magnetic separator (Invitrogen) was used to recover the bead-bound cells. The recovered cells were washed with DMEM-20%, suspended in 10 ml of complete culture medium [DMEM containing 20% FCS, supplemented with 100 μg/ml porcine heparin (Sigma), 100 μg/ml EC growth stimulant (ECGS, BioRad, Hercules, CA, USA), nonessential amino acids (Thermo, Rockford, IL, USA), sodium pyruvate (Sigma), l-glutamine (Gibco), and antibiotics(Gibco)], and then plated in a single gelatin-coated 75-cm^2^ tissue culture flask. After overnight incubation, the nonadherent cells were removed, the adherent cells washed with Hanks’ balanced salt solution (Gibco), and 10 ml of fresh complete media was added. Cultures were fed routinely on alternate days with fresh complete culture medium. When the cells reached 70 to 80% confluence, they were detached with warm trypsin-ethylenediaminetetraacetic acid (GE Healthcare, Logan, UT, USA) to generate a single cell suspension. The cells were pelleted and then resuspended in 2 ml of DPBS and sorted for a second time using ICAM-2-coated (BD) beads (15 μl/ml of cells). The bead-bound cells were washed and plated in complete culture medium and passaged further at a 1:2 ratio. Confluent monolayers of multiple preparations (3 to 4 MLEC isolates) were used at passages 1 to 3 for this study. The cells were allowed to reach confluence and passaged 1–2 times more before experiments were performed. Purity of the cell population was assessed by CD146 FITC (Miltenyi Biotec, Bergisch Gladbach, Germany) staining and flow cytometric analysis with FACSCanto (BD, data not shown).

### Proliferation assay

Cellular proliferation of isolated ECs was analyzed by MTT assay. 96-well plates were coated with fibronectin (BD), collagen (Corning, NY, USA), and gelatin (Sigma). Cells were plated at a cellular density of 1 × 10^4^ cells per well in 100 μl of medium. After 24 h, 48 h, or 72 h incubation all wells received 20 μl of the MTT reagent (Promega, Madison, WI, USA) and were incubated for 2 h at 37 °C. Color intensity was measured in a plate reader (BMG Labtech) at 490 nm.

### Measurement of impedance-based wound healing of confluent EC cultures

Wound healing was determined in EC using the electric cell-substrate impedance sensing (ECIS) system (Applied Biophysics, Troy, NY, USA) as described previously^64^. 1.2 × 10^4^ EC from WT and KO mice were grown with 20% fetal bovine serum (FBS) culture medium on coated ECIS electrode arrays (8W1E). The impedance fluctuations of cell attachment and spread were continuously monitored. Impedance measurements were analyzed at 5 minute intervals and verified that confluence was achieved. At confluence, wounding of EC was achieved using a 1400 μA signal at 60 kHz for a duration of 20 sec. Application of this field results in a rapid drop in the impedance of cell layers due to the death of the cells on the electrode. Impedance increases as cells migrate from the perimeter of the electrode inward to replace the killed cells. The slope of the resulting line would be proportional to the migration capabilities of the cells.

### Transwell migration assay

In addition, cell migration assays were performed using transwells. 12-well format plates with 8 μm pore size inserts (Corning) were coated as above. 5 × 10^4^ cells in 200 μl of media without FBS were added to the upper compartment. 500 μl of DMEM with 20% FBS containing ECGS were added to the lower compartment. Cells were incubated in transwell plates at 37 °C and 5% CO_2_. After 48 h the insert was taken out. Cells on the lower side of the insert filter were quickly fixed in 5% glutaraldehyde (Sigma) for 10 minutes, then stained with 1% crystal violet (Sigma) in 2% ethanol for 20 minutes. Images of the lower side of the filter were recorder under a microscope (Leica CTR4000, Wetzlar, Germany) and the number of cells was counted.

### RNA extraction

Total RNA was extracted from MLEC samples using TRIzol (Invitrogen), purified using an RNeasy Mini kit (Qiagen, Valencia, CA, USA), and treated with DNase I (Qiagen) following manufacturer’s instructions.

### cDNA library preparation and ultrasequencing

Library preparation and ultrasequencing were performed following Illumina’s (San Diego, CA, USA) protocols. Most reagents were also from Illumina.

First, the integrity and quality of total RNA were assessed with the Experion Automated Electrophoresis system (BioRad). Then, mRNA was isolated from 1 mg of total RNA using poly-T oligo-attached magnetic beads. This mRNA was fragmented into pieces of approximately 200 bp using divalent cations under elevated temperature. The cleaved RNA fragments were reverse transcribed into first strand cDNA using reverse transcriptase and random primers. Next, the second strand was synthesized using DNA polymerase I and RNAse H. These double-stranded cDNA fragments were end-repaired by T4 DNA polymerase and Klenow DNA polymerase, and phosphorylated by T4 polynucleotide kinase. The cDNA products were incubated with Klenow DNA polymerase to generate 3′ Adenine overhangs, therefore allowing ligation to Illumina indexing adapters to the double stranded cDNA ends. The adapter-ligated products were purified with Ampure XP magnetic beads (Agencourt Bioscience Corporation, Beverly, MA, USA) and libraries were amplified by 15 cycles of PCR with Phusion DNA polymerase (Finnzymes Reagents, Vantaa, Finland). Constructed libraries were validated and quantified using BioRad’s automated electrophoresis system Experion and qPCR respectively. Pools of 6 indexed libraries were mixed (multiplexed) at equimolar ratios to yield a total oligonucleotide mix concentration of 10 nM. Finally the resulting libraries were sequenced on the Genome Analyzer IIx platform (Illumina) to generate 150 bp single reads. Six pooled indexed libraries were sequenced in each flow cell lane.

### Bioinformatic analysis

Raw reads were filtered to eliminate Illumina adapters with Cutadapt software (http://cutadapt.readthedocs.org/en/stable/index.html) and read quality was assessed with FastQC (v0.10.1; http://www.bioinformatics.babraham.ac.uk/projects/fastqc/). Clean reads were mapped against *Mus musculus* GRCm38.71 reference genome by using Tophat2 tool (version 2.0.6; http://ccb.jhu.edu/software/tophat/index.shtml)^65^. Transcriptome analysis was performed with SeqSolve (Integromics, v5.5 https://www.integromics.com/products/genomics/ngs/). Differential Expressed Genes were determined according to their FPKMs values at FDR 0.05 and 0.1. The data discussed in this publication have been deposited in NCBI’s Gene Expression Omnibus and are accessible through GEO Series accession number GSE84345 (https://www.ncbi.nlm.nih.gov/geo/query/acc.cgi?acc=GSE84345).

### Endothelial function during tumorigenesis

This study exploited the established B16-BL6 model of lung metastatic tumor growth^66^. Briefly, 300 μl of PBS containing 5 × 10^5^ freshly harvested B16-BL6 cells were i.v. injected into AM^EC-KO^ and WT mice. After 21 days, mice were sacrificed, and the lungs were removed and weighed. Lungs were inflated with buffered formalin and paraffin-embedded. Sections (3 μm-thick) were stained with hematoxilin eosin. Several parameters were quantified: lung weight, number of tumors observed by the naked eye, diameter of the largest tumor, and number of tumors per histological section.

### Permanent focal brain ischemia model

Focal brain ischemia was carried out in mice by permanent middle cerebral artery (MCA) occlusion (MCAO) as previously described^30^. Briefly, an incision perpendicular to the line connecting the lateral canthus of the left eye and the external auditory canal was made to expose and retract the temporalis muscle. A burr hole was drilled and the MCA was exposed by cutting and retracting the dura. The MCA was elevated and cauterized, thus producing a permanent MCA occlusion (pMCAO). Following surgery, subjects were returned to their cages and allowed free access to water and food. Two groups of animals were used, corresponding to AM^EC-KO^ and their WT counterparts. Two days after pMCAO, animals were killed by an overdose of sodium pentobarbital to assess infarct volume outcome. The brain was removed and cut into seven 1 mm-thick coronal brain slides (Brain Matrix, WPI, UK) and stained with 2,3,5-triphenyltetrazolium chloride (1% TCC in 0.1 M phosphate buffer, Sigma). Infarct volumes were calculated sampling each side of the coronal sections with a digital camera (Nikon Coolpix 990, Tokyo, Japan), and the images were analyzed using Image J free software (The NIH, Bethesda, MD, USA). The area of infarct, which is unstained, was determined by counting the pixels contained within the outlined regions of interest and expressed in square millimeters. Infarct volumes (in mm^3^) were integrated from the infarct areas over the extent of the infarct calculated as an orthogonal projection. All animals displayed infarcts after the occlusion procedure, which included the cortex, subcortex, and striatum, depending on the intensity of the lesion.

### Statistical analysis

All data were analyzed with GraphPad Prism 5 software and were considered statistically significant when p < 0.05. Values are expressed as means ± SEM. Normally distributed data were evaluated by ANOVA followed by the Dunnet’s (Bonferroni) post-hoc test while data not following a normal distribution were analyzed with the Kruskal-Wallis test followed by the Mann-Whitney U test.

## Additional Information

**How to cite this article**: Ochoa-Callejero, L. *et al*. Lack of adrenomedullin in mouse endothelial cells results in defective angiogenesis, enhanced vascular permeability, less metastasis, and more brain damage. *Sci. Rep.* **6**, 33495; doi: 10.1038/srep33495 (2016).

## Figures and Tables

**Table 1:** Blood pressure in adult WT and AM^EC-KO^ mice.

	WT	KO	*p*
Systolic blood pressure (mm Hg)	134.67 ± 2.29	126.20 ± 1.64	<0.001***
Diastolic blood pressure (mm Hg)	92.37 ± 2.14	91.03 ± 1.46	0.05
Pulse (betas/min)	672.83 ± 12.75	687.38 ± 5.38	0.33
Blood flow rate (mL/min)	22.04 ± 0.45	22.27 ± 0.52	0.82
Tail blood volume (μl)	20.50 ± 0.60	26.06 ± 0.89	<0.001***

Blood pressure parameters as measured with the CODA system in wild type (WT) and knockout (KO) mice (n = 8 per group). Values are mean ± SEM of all valid measurements. Systolic blood pressure was lower in AM^EC-KO^ than in WT mice. Tail blood volume was higher in AM^EC-KO^ than in WT mice.
