# Author Correction: Lack of adrenomedullin in mouse endothelial cells results in defective angiogenesis, enhanced vascular permeability, less metastasis, and less brain damage

**DOI:** 10.1038/s41598-020-62788-0

**Published:** 2020-04-08

**Authors:** Laura Ochoa-Callejero, Andrea Pozo-Rodrigálvarez, Ricardo Martínez-Murillo, Alfredo Martínez

**Affiliations:** 1 Oncology Area, Center for Biomedical Research of La Rioja (CIBIR), C/Piqueras 98, 26006 Logroño, Spain; 2 Department of Molecular, Neurovascular Research Group, Cellular and Developmental Neurobiology, Cajal Institute, Av. Doctor Arce 37, 28002 Madrid, Spain

Correction to: *Scientific Reports* 10.1038/srep33495, published online 19 September 2016

This Article contains an error in the title of the paper.

“Lack of adrenomedullin in mouse endothelial cells results in defective angiogenesis, enhanced vascular permeability, less metastasis, and more brain damage”

should read:

“Lack of adrenomedullin in mouse endothelial cells results in defective angiogenesis, enhanced vascular permeability, less metastasis, and less brain damage”

